# ﻿An unexpected new tree species from Gansu, China: *Illiciumgansuense* (Schisandraceae)

**DOI:** 10.3897/phytokeys.230.102754

**Published:** 2023-08-25

**Authors:** Zengfu Bai, Zhihua Zhang, Xuelin Chen, Ji Zhang

**Affiliations:** 1 College of Life Sciences, Northwest Normal University, Lanzhou, 730070, Gansu, China Northwest Normal University Lanzhou China; 2 Institute of New Rural Development, Northwest Normal University, Lanzhou, 730070, Gansu, China Northwest Normal University Lanzhou China

**Keywords:** Austrobaileyales, basal angiosperms, Gansu, Giant Panda National Park, Illiciaceae, Yuhe area

## Abstract

We describe the newly-discovered species *Illiciumgansuense* (Schisandraceae), discovered in the Yuhe area of Giant Panda National Park, Gansu, China. Morphologically, *I.gansuense* resembles *I.ternstroemioides* and *I.arborescens*. However, the new species can be distinguished by its smaller leaf size, the larger number of tepals, tepal margin ciliate, and distinct flowering and fruiting seasons.

## ﻿Introduction

The genus *Illicium* L., which belongs to the family Schisandraceae (APG IV 2016), consists of 35 species distributed across the southeastern United States, Mexico, the West Indies (five species), and eastern Asia (approximately 30 species) (Keng 1993). Linnaeus (1759) published the 10^th^ edition of Systema Naturae, which included the first named species of *Illicium* (*I.anisatum* L.) based on Kaempfer’s monograph. In 1947, Smith published the first comprehensive study of the genus *Illicium*, “The Families Illiciaceae and Schisandraceae”, which divided the genus into two subgenera based on perianth morphology: 1) I.subgen.Illicium with thin, membranaceous and narrowly oblong or ligulate inner perianth segments; and 2) I.subgen.Cymbostemon with carnose to papyraceous and usually ovate to suborbicular inner perianth segments (Spach 1839; Smith 1947; Lin 1997). Both Hao’s and Morris’ results suggested that the previous division based on perianth morphology wasn’t monophyletic (Hao et al. 2000). Morris et al. (2007) utilized a distinguishable seed character to reflect their evolution history: I.sect.Cymbostemon contains seven species with a hilar rim around seed hilum; and I.sect.Illicium consists of all the other species which do not have a hilar rim.

China, especially the southwest and southeast parts of the country, is home to many species of *Illicium* species. In particular, the species *I.henryi* Diels. is known to occur only in southern Gansu Province.

*Illicium* has considerable economic value, with *I.verum* Hook.f. being particularly valuable domestically in China and exported worldwide. The fruits of *I.verum* are used as a spice, and the leaves and fruits are steam distilled to create an aromatic oil (star anise oil) for use as a flavoring, as well as a therapeutic agent in traditional Chinese medicine (TCM). Other species of *Illicium* are used ornamentally or as a source of fine wood for furniture.

While surveying plants in the Yuhe area of the Giant Panda National Park, Gansu, in October 2020, we discovered an unknown species of *Illicium*. Based on field surveys, morphological and phenological studies, and taxonomic literature reviews, we concluded that this species should be included in I.sect.Cymbostemon (Spach) A.C.Smith (Smith 1947). However, because the specimen differed from other members in the section, we named and established the specimen as a new species (*I.gansuense*), as described herein.

## ﻿Material and method

All morphological data were obtained from field observations carried out in the Yuhe Area of the Giant Panda National Park, Longnan City, Gansu Province, eastern China. Plants were photographed with a Nikon D750 digital camera. Digital specimens were deposited at the IBSC, KUN, PE, IBK, and WUK herbaria through the Chinese Virtual Herbarium (https://www.cvh.ac.cn/). Physical specimens were deposited at the NWTC Herbarium. All terminology used in the present study is in accordance with the Flora of China (Lu and Rabeler 2001), as well as the Plant Photo Bank of China (PPBC) and the Chinese Field Herbarium (CFH). The conservation status of the new species was assessed according to the standards of the IUCN (2012).

## ﻿Taxonomic treatment

### ﻿ Illicium gansuense

Taxon classificationPlantaeAustrobaileyalesSchisandraceae

Z.F.Bai & Xue L.Chen sp. nov.

01EEA136-65E6-588B-8258-B61FC86664C6

urn:lsid:ipni.org:names:77325768-1

Figs 2 , 3 , 4

#### Type.

China. Gansu: Longnan City, Yuhe Area of Giant Panda National Park, altitude ca. 1200 m, 4 April 2020, *Zengfu Bai & Xuelin Chen 2020001* (***holotype***: NWTC!; ***isotype***: NNBG!).

#### Diagnosis.

*Illiciumgansuense* is similar to *I.ternstroemioides* and *I.arborescens* in overall form, leaf characters, red flowers, location, and population density. *Illiciumgansuense* can be distinguished from *I.ternstroemioides* and *I.arborescens* based on leaf-blades size (7–12 × 1.8–3.5 cm in *I.gansuense* vs. 7–13 × 2–5 cm in *I.ternstroemioides* vs. 6–12 × 2–4.5 cm in *I.arborescens*), tepal number and pubescence (10–17 tepals with ciliate margins vs. 10–14 tepals with glabrous margins vs. 14–21 tepals with glabrous margins), number of carpels (10–13 vs. 12–14 vs. 12–16), number and size of the stamens (23–27, 2–3 mm long vs. 22–30, 1.8–3.4 mm long vs. 39–41, 2–3 mm long), and ovary length (1–1.5 mm long vs. 1.3–2.5 mm long vs. 1–1.8 mm long). (Table 1).

**Table 1.** Morphological, geographic, and phenological comparison of *Illiciumgansuense*, *I.ternstroemioides*, and *I.arborescens*.

Trait	*I.gansuense*	*I.ternstroemioides*	*I.arborescens*
**Leaf blades**	7–12 × 1.8–3.5 cm	7–13 × 2–5 cm	6–12 × 2–4.5 cm
**Tepals**	10–17, margin ciliate	10–14, margin glabrous	14–21, margin glabrous
**Carpel**s	10–13	12–14	12–16
**Stamens**	23–27, 2–3 mm long	22–30, 1.8–3.4 mm long	39–41, 2–3 mm long
**Ovaries**	1–1.5 mm long	1.3–2.5 mm long	1–1.8 mm long
**Distribution**	Gansu (eastern China)	Fujian, Hainan (southeastern China)	Taiwan
**Flowering time**	March–April	January–August	January–April

#### Description.

Trees 4–12 m tall, whole plant with an aniseed aroma. Trunk 22.5 cm diam. at chest height, outer bark grayish-brown, with irregular longitudinal cracks; canopy tower or conical and branches are dense and spread horizontally; twigs pubescent, perules ca. 3 × 2 mm, ovoid, yellowish-brown, caducous, margins finely ciliate. Leaves clearly spirally-alternate to pseudoverticillate (clustered in sets of 2–5 at the apex of twigs); petioles 8–12 mm long, 1–2 mm diam.; blades 7–12 × 1.8–3.5 cm, oblanceolate, coriaceous, translucent oil spots visible against the light, adaxially dark to medium green, glossy, abaxially light green, base cuneate, margin glabrous, apex acuminate; midvein adaxially slightly impressed, abaxially prominently round, lateral veins pairs 6–9, inconspicuous. Inflorescences 1-flowered, but flowers sometimes clustered in groups of 2–6 at the apex of branches, axillary, pedunculate; peduncle 8–14 mm long, 2 mm diam., brown, bracteoles 2–4 × 2–3 mm, ovate. Flowers 12–18 mm diam., bisexual, androgynous scented, anthesis diurnal; floral buds 5–10 × 3–6 mm, ovoid, brown; pedicels 5–12 mm long, 2 mm diam., brown; tepals 10–17, in 2–3 whorls, outer whorl with 5–7 tepals, 6–8 × 5–7 mm, ovate, sepaloid, yellowish-green, base round, margin red, ciliate, apex acuminate or obtuse, inner whorls with 8–10 tepals, 8–12 × 4–8 mm, widely ovate to widely obovate to widely elliptic, petaloid, fleshy, red, base broadly cuneate, margin ciliate, apex acuminate; stamens 23–27, in 2–3 whorls, 1.3–3.5 mm long, filament 0.3–1.5 mm long, stout, widely obovoid to widely ellipsoid, pink, connective truncate to emarginate, pink, anther 1–2 × 0.6–1 mm, introrsely rimose, pollen grains trisyncolpate, blackish-brown *in vivo*; carpels 10–14, 3–5.5 × 1.6–2 mm, ovaries 1–1.5 mm long, stigmatic crest 1.3–1.8 mm long, slightly longer than the ovary, subulate. Follicetum 12–16 × 4–7 mm; peduncle 1–1.5 cm long; follicles 10–13, 15–25 × 5–8 mm, 2–4 mm thick, woody, dark brown, apex aristate due to the persistent and hardened stigmatic crest, 3–6 mm long, slightly curved at apex. Seeds 4.5–6 × 4–5 mm, 1.5–2.5 mm thick, ovoid, testa smooth, brown.

#### Distribution and habitat.

**Figure 1.**
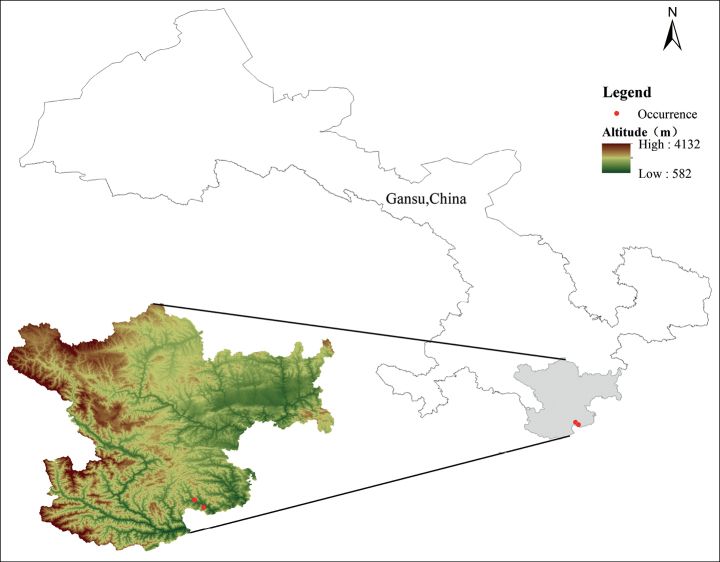
Distribution of *Illiciumgansuense*.

Currently, only one population of *I.gansuense* has been identified in Yuhe Town, Longnan City, southern Gansu Province. This area is characterized by a northern subtropical subhumid climate and a mountainous terrain containing high peaks and steep slopes. Specimens of *I.gansuense* were found growing in a deciduous broadleaf forest at an elevation of 1200 m. The dominant species of this forest community include *Trachycarpusfortunei* (Hook.) H.Wendl. (Arecaceae), *Cinnamomumseptentrionale* Hand.-Mazz. (Lauraceae), *Linderaaggregata* (Sims) Kosterm. (Lauraceae), and *Deyeuxiaeffusiflora* Rendle (Poaceae). (Fig. 1)

#### Phenology.

Flowering from March to April, fruiting from May to November.

#### Conservation status.

There is only one known location, and fewer than three individuals of *I.gansuense* were found during our fieldwork in the Yuhe area of Giant Panda National Park in 2020 and 2022. However, investigations of the natural distribution of this species are insufficient. According to the IUCN Red List criteria (2019), this new species is better assessed as Data Deficient (DD; criteria B1ab(i–v) + 2ab(i–v)).

**Figure 2.**
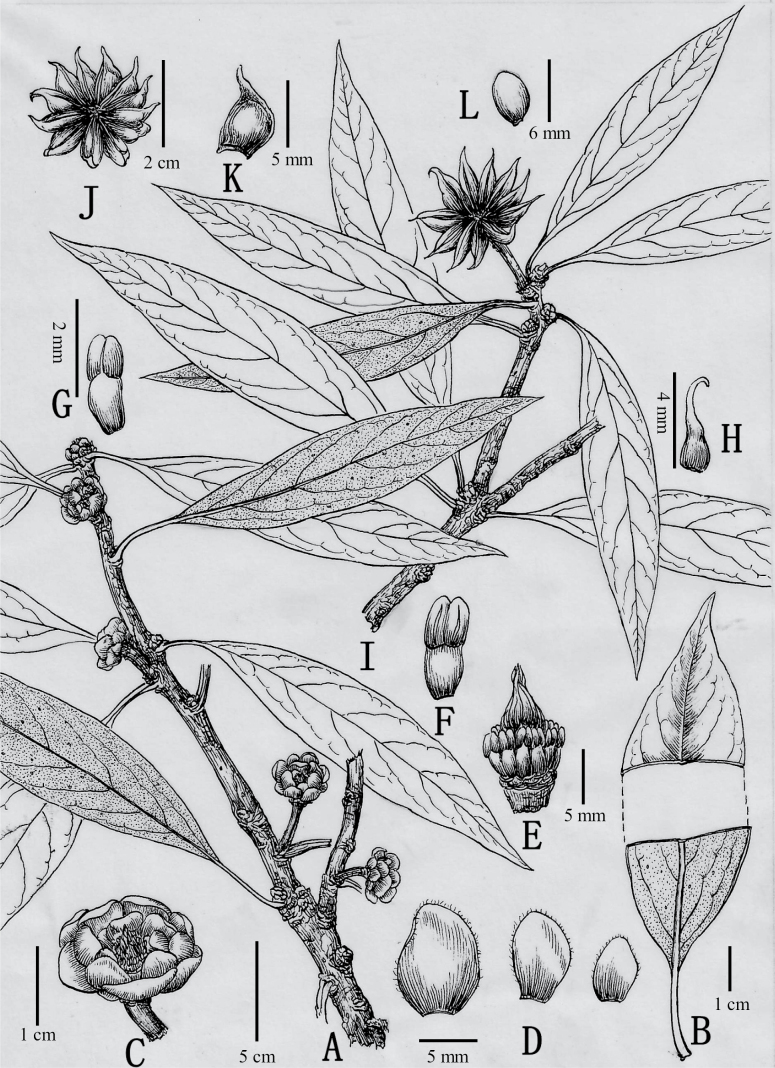
*Illiciumgansuense* Z.F.Bai & Xue L.Chen **A** flowering branch **B** adaxial and abaxial leaf surface **C** flower **D** tepals **E** removal of tepals showing gynoecium and stamens **F, G** stamens, dorsal and ventral views **H** carpel **I** fruiting branch **J, K** fruits **L** seed. (Drawn by Jianlu Bai based on type specimen).

#### Etymology.

The specific epithet ‘*gansuense*’ refers to a province in eastern China. 甘肃八角 (gān sù bā jiǎo) is suggested as a suitable Chinese name for it.

**Figure 3.**
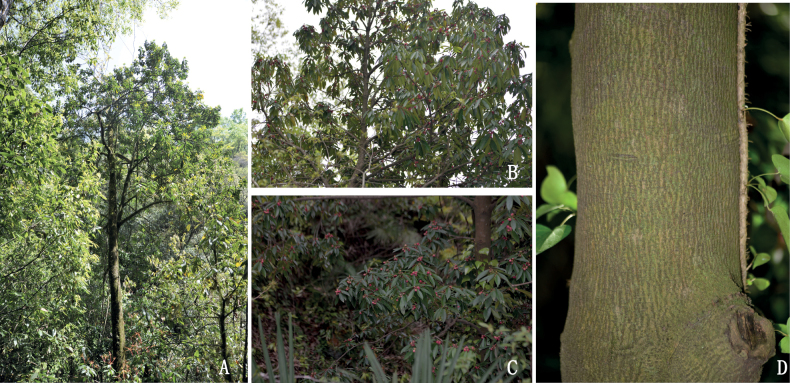
*Illiciumgansuense* Z.F.Bai & Xue L.Chen **A** habitat **B, C** flowering branch **D** bark.

#### Notes.

*Illiciumgansuense* is similar to *I.ternstroemioides* and *I.arborescens* in overall form, that are all trees, leaf characteristics, flower color, location, and population density. However, as noted above, the three species can be distinguished according to both morphological features and distribution. Specifically, in relation to *I.ternstroemioides* and *I.arborescens*, *I.gansuense* is characterized by smaller leaf blades, tepals with ciliate margins (rather than glabrous), fewer carpels, and smaller ovaries. Additionally, *I.gansuense* blooms from March to April and *I.arborescens* blooms from January to April. (Table 1).

**Figure 4.**
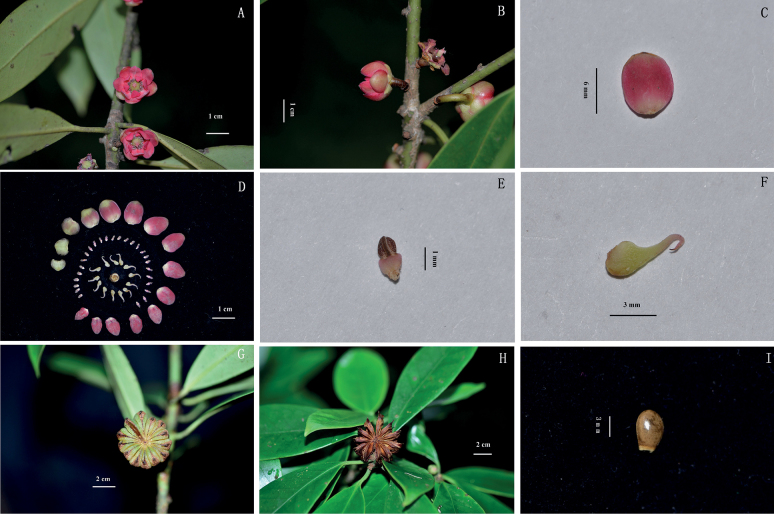
*Illiciumgansuense* Z.F.Bai & Xue L. Chen **A** flower at front view **B** flower at side view **C** the largest tepals **D** all parts of flower **E** stamen **F** follicle **G** fruit at front view **H** fruit at side view **I** seed. Photographed by Zengfu Bai.

## Supplementary Material

XML Treatment for Illicium gansuense

## ﻿Additional information

### Data availability

All of the data that support the findings of this study are available in the main text.
